# Promoting positive perceptions of and motivation for research among undergraduate medical students to stimulate future research involvement: a grounded theory study

**DOI:** 10.1186/s12909-020-02112-6

**Published:** 2020-06-26

**Authors:** Belinda W. C. Ommering, Marjo Wijnen-Meijer, Diana H. J. M. Dolmans, Friedo W. Dekker, Floris M. van Blankenstein

**Affiliations:** 1 Center for Innovation in Medical Education, Leiden University Medical Center, Hippocratespad 21, Zone V7-P, PO Box 9600, Leiden, The Netherlands; 2 Technical University of Munich, TUM School of Medicine, TUM Medical Education Center, Munich, Germany; 3 Department of Educational Development and Research, School of Health Professions Education, Maastricht University, Maastricht, The Netherlands; 4 Department of Clinical Epidemiology, Leiden University Medical Center, Leiden, The Netherlands

**Keywords:** Undergraduate research, Perceptions of research, Motivation, Physician-scientist, Grounded theory

## Abstract

**Background:**

Research is of great value to make advancements within the medical field and, ultimately, offer the best possible patient care. Physician-scientists are key in contributing to the development of medicine, as they can bridge the gap between research and practice. However, medicine currently faces a physician-scientist shortage. A possible solution to cultivate physician-scientists is to engage medical students in research in early phases of medical school. Evidence-based strategies to stimulate positive perceptions of and motivation for research among students could help to enhance research engagement. Consequently, understanding of students’ perceptions of and motivation for research is needed. Therefore, this study aimed to identify conditions under which students develop positive perceptions of and motivation for research by answering the following sub-questions: 1) how do first-year medical students perceive research? and 2) which factors contribute to motivation or demotivation for conducting research?

**Methods:**

We conducted a qualitative study with individual interviews using a grounded theory approach, involving 13 purposively sampled first-year medical students at Leiden University Medical Center.

**Results:**

Our results suggest that first-year students are already able to identify many aspects of research. Students elaborated on the relevance of research for professional practice and personal development. Furthermore, our results suggest a relationship between perceptions of and motivation for research. Some perceptions were identical to motivating or demotivating factors to conduct research, like the relevance of research for practice and performing statistics respectively. Other motivating factors were, among others, acknowledgment, autonomy, and inspiring role models. Demotivating factors were, among others, lack of autonomy and relevance, and inadequate collaboration.

**Conclusions:**

Our results contribute to the idea that perceptions of research are related to motivation for research, which offers possibilities for interventions to promote motivation for research by making use of student perceptions of research. Consequently, practical implications to stimulate research engagement in early phases of medical school are provided. Moreover, the results contribute to existing motivational theories like Theory of Planned Behavior and Self-Determination Theory within this specific domain.

## Background

Scientific research is of great value to make advancements within the medical field and, ultimately, offer the best possible patient care. In order to practice evidence-based medicine, all physicians should be aware of the newest developments and involve scientific knowledge (e.g. research) in clinical decision making [1–4]. In addition, physicians who actually conduct research (i.e. physician-scientists) are needed as well. Physician-scientists devote a substantial amount of their time to both clinical practice and conducting research, and are thereby key in bridging the gap between science and practice [5–7].

Unfortunately, the medical field is facing a global shortage of physician-scientists. The current physician-scientist workforce is aging and a decrease in interest to pursue a scientific career is visible in the United States, Canada, and Europe. Recent literature stresses the urgent need to counteract this decline in the physician-scientist workforce [1, 8, 9].

Engaging students in research during early phases of medical school could help to acquaint students with research, trigger enthusiasm, and direct more students towards a physician-scientist career [1, 7, 10, 11].

In order to draw pre-clinical students into research during medical school, knowledge and understanding is needed on how they perceive research and the importance of conducting research for clinical practice. The question arises to what extent these young medical students already comprehend what it is to conduct research and how this relates to clinical practice. Additionally, it is important to know what motivates or demotivates students in their consideration to conduct research [12].

Studies investigating perceptions of and motivation for research among pre-clinical medical students are scarce. Few studies have focused on perceptions of research and its importance for practice among medical students. For instance, there is evidence that students do not realize the importance of research for clinical practice until the clinical phase of medical training, when they encounter real life problems in patient care [13]. This is in line with previous findings indicating that undergraduate students have a narrow perspective of research and are not aware of the connection between research and practice [14–16]. Nel and colleagues surveyed medical students at the University of Capetown, and found that 61% of the students had positive attitudes towards research [17]. However, they did not identify the nature of these attitudes. Some of the prior studies also examined motivation for research and suggested that most medical students are motivated to pursue research, but foresee many difficulties and barriers at the same time [15–17]. In one of our earlier studies, we did find students to be highly motivated for research when entering medical school. These results also indicated that pre-clinical students’ beliefs about the value of research were important to influence research motivation [18]. In turn, research motivation was related to actual research involvement among undergraduate medical students [19]. This implies that insights into how beginning medical students perceive research could be of great value in directing more medical students towards a physician-scientist career. However, the few conducted studies in this area did not mainly focus on early stages of medical training.

In sum, there seems to be insufficient knowledge about how pre-clinical medical students beginning their medical studies perceive research and how they could be motivated to conduct research. Furthermore, most of the aforementioned studies had a quantitative approach. Since the aim is to engage medical students in research in early phases of medical school, deeper understanding of pre-clinical students’ perceptions and motivation regarding research is valuable, for which a qualitative methodology seems imperative. This could help to identify how positive perceptions of and motivation for research can be promoted early on in medical training. In turn, these insights could help to determine possible interventions and the implementation of evidence-based strategies to enhance interest in research among medical students, thereby cultivating future generations of physician-scientists.

Therefore, this study uses a qualitative grounded theory approach to gather in-depth knowledge on how educators can create conditions under which pre-clinical medical students develop positive perceptions of and motivation for research during early phases of medical school, by answering the following two sub-questions: 1) how do first-year medical students perceive research? And 2) which factors contribute to motivation or demotivation for conducting research?

## Methods

### Context

This study was conducted among one cohort of first-year medical students at Leiden University Medical Center (LUMC). The Netherlands has eight medical schools, which all developed their educational program in line with the Dutch National Blueprint for Medical Education. The schools offer six years of undergraduate and graduate medical education. In the Netherlands, most students start medical school immediately after graduating from secondary school, at the age of 18–19 years [20]. Consequently, first-year medical students are relatively young and lack any research-related experience [21]. In this study, students’ only prior experiences with research were a two-week course at the start of their medical training. In this course, students conducted a small research project and were actively involved in gathering and processing data, formulating their own research question, analyzing data and writing a two-page research report [22].

### Research team

The research team comprised of five researchers from different backgrounds. BO is a PhD-candidate in medical education, with a master’s degree in Pedagogical Sciences. FB is senior researcher in medical education. MWM is full professor in medical education. DD is full professor of innovative learning arrangements. FD is full professor in undergraduate research in medical education and clinical epidemiology. BO, MWM, DD and FB have experience with qualitative research approaches and analysis.

### Design

We established our research within an interpretivist paradigm, emphasizing the subjective nature in understanding human experiences and creation of reality. According to this paradigm, reality is socially constructed and truth is not grounded within one single objective reality. Rather, there may be multiple ways by different individuals to interpret a single construct or phenomenon [23]. Within the interpretivist paradigm, there is an emphasis on valuing the unique views of every individual. Consequently, we used a qualitative grounded theory approach as this eminently suits the aim to create deeper understanding of the unique perceptions of each individual in our study, including purposive sampling and constant comparison. Data was iteratively collected and coded, until saturation and consensus among the first and last author (BO & FB) was reached. We used semi-structured individual interviews to identify and elucidate students’ perceptions of and motivation for research.

### Participants

All first-year students were informed about the study before the start of a lecture. Students were given the opportunity to apply for participation in this study by signing a registration list, which in total 22 students did. Thereafter, a purposive sampling method (i.e. selective sampling based on the researchers judgment when choosing participants for the study) was applied, aiming to include different types of students in our sample. In our earlier study, all first-year students were surveyed at the beginning of medical school and reported on their research motivation and self-efficacy [18]. Data of the 22 students who signed the registration list from this questionnaire was used in the sampling procedure, aiming to include diverse types of first-year students scoring differently on intrinsic and extrinsic motivation for research, and research self-efficacy. Furthermore, we aimed to include students who were both interested and not interested in entering an extracurricular research-based Honors program in the second year of education. Lastly, gender and age were included in the selection process.

Between March 2017 and September 2017, BO approached the purposive sampled students by e-mail. Data collection and analysis were performed in an iterative manner, eventually resulting in a total of 13 first-year medical students who were invited and all agreed to participate in our study. This study included 10 female (76.9%) and 3 male (23.1%) students, which is representative for the male/female distribution in the whole cohort (i.e. the total number of first-year students starting medical training in 2016). Students were 18 to 20 years, with a mean age of 19.3 years.

### Data collection

BO and FB developed an interview guide (Appendix 1), which was checked on followability by discussing it within the research team. BO conducted all interviews, which were audio-recorded and transcribed verbatim line by line. Additionally, a summary was made of the content of the interview, which was then sent to the participant for member checking (i.e. participant check on accuracy). All participants agreed on the content. When participants’ quotes were used to illustrate results, participants were again approached to ask for their permission. Every participant agreed on the use of their quotes.

### Data analysis

Data analysis was performed alongside data collection in an iterative manner. All interviews were independently coded by BO and FB using a grounded approach. BO and FB discussed their initial findings in the process of analysis, to reach consensus, and built a codebook (i.e. overview of all themes; Appendix 2). Three types of coding as described by Strauss & Corbin were used: open, axial, and selective [24].

Fragments or sentences of the transcript were coded with an ‘in vivo approach’ (i.e. open coding), followed by interpretative analysis to create overarching categories (i.e. axial coding). Lastly the overarching categories were checked, subsequently followed by the creation of higher-order themes (i.e. selective coding). After the stage of analysis was completed and a codebook with higher-order themes was created, MWM checked followability of the steps that were made in this process. In addition to the completed analysis, BO, FB and MWM independently coded two interviews with the new codebook to test its reliability. All interpretations were then discussed within the entire research team. Data analysis was supported by Atlas-ti 8.0 software (Atlas.ti, GmbH, Berlin, Germany).

### Ethical approval

Students gave verbal consent on the audio-recording before the interview and signed an informed consent form after the interview. In compensation for their time, students received a gift certificate of €7.50 to spend in the lunchroom of the LUMC. This study was approved by the Educational Institutional Review Board of the LUMC (IRB reference number: OEC/OG/20180508/2).

## Results

We conducted 13 interviews, of which the length varied between 25 and 42 min. Inductive thematic saturation (i.e. no new themes emerged) and theoretical saturation of the themes (i.e. no additional data to develop a theme was found, as the researcher sees similar instances over and over again) [25] was reached after 11 interviews, after which we conducted two last interviews to check saturation. Because of the rich data, not all subthemes are discussed in detail. An overview of all themes can be seen in Appendix 2.

### How do first-year medical students perceive research?

Five higher-order themes emerged: research processes, research goals, research characteristics, research topics, and research requirements.

Students mainly focused on several parts of the *research process*, mentioning creating research questions, choosing a method, gathering data, processing data, creating results, drawing conclusions, and reporting outcomes. On the one hand, some students had the perception that research consisted of single, specific parts, reflecting a relatively narrow definition of research.

[Research is] the whole day in the lab or doing your best to persuade people to participate in your research. – S1

On the other hand, in some cases students did connect multiple phases of conducting research, creating a bigger picture of what the process of research entails.

[Research] exists out of, for a large part, pre-work; thinking about what you want to study, how you are going to do that, methods, participants or something like that. And if you have devised the entire research, then you will carry it out, for instance by interviewing like this I think, it depends on the kind of research you’re performing, if you will do tests or something like this, and then thereafter it exists out of processing all your data, of course, drawing conclusions from it, and writing an article about it. – S12

However, students tended to focus on more than only these concrete aspects of doing research. They also mentioned *research goals*, reflecting on the importance of research for society and healthcare in general. For instance, the valuable role research plays in creating new knowledge or refining existing knowledge, and thereby the improvement of understanding in general.

Some fundamental studies are done for understanding, a sort of, contribution to the general understanding of how something works. – S1

Furthermore, students had more specific goals of research in mind as well, emphasizing the medical context. In particular, students elaborated on developing and improving medicines or illness treatments, but also on improving the organization within the whole hospital. Moreover, students also discussed the role research could play in improving education, which in turn helps to educate and deliver better physicians.

I think that with research, on the one hand, we can gather more knowledge on the emergence of diseases and the human body, but on the other hand we can treat these diseases better or even find a cure. But I also think that, within medical healthcare, there also exists research into, for instance, collaboration between people and the best way to shape a hospital, or the best way to work within teams. – S7

Perceptions of research were also illustrated in different *characteristics* students assign to research. Students tended to concentrate on negative aspects, like the hard and intensive character of research. The idea that conducting research is hard is mostly related to the lack of or difficulty in finding results.

I think you need to have perseverance [to conduct research], because nine out of ten times you will get a result you actually did not want to have. – S13

Moreover, research is seen as an intensive and complex activity in which different tasks need to be combined, the researcher has many different appointments and several obligations like following rules and administrative work.

You need to be able to make appointments, very many appointments, and you need to make sure to work on your own research, you must write a text, all that taken together, you need to arrange that in a good way to prevent double appointments and to prevent that, because of all the appointments, you can’t write. So, yeah… it seems like a busy thing to me. – S4

Students also commented on *research topics,* namely healthcare, prevention and organization.

You have health-promoting, which predominantly focuses on prevention areas of research, but you also have research into different diseases and mechanisms. But I think that you can also study the way an organisation works and how they collaborate within medical contexts. – S3

The last higher-order theme that emerged, is one that is not directly linked to research itself. The first-year medical students also described *research requirements*, illustrating conditions that researchers must meet in order to actually perform their research. Students emphasized the importance of collaboration, finance, and ethical approval.

A researcher is not only doing the research itself, but also busy with financing, arranging to be able to work with other people. I think that next to the research itself, research entails more, a researcher does more than just the research on its own. – S7

### Which factors contribute to motivation for conducting research?

Students reported motivators for research from the perspective of personal benefit. For instance, they would be motivated to do research because it would contribute to their *personal development*. Students mentioned a lack of academic training and challenge in the curriculum, and the need to delve into certain topics instead of just learning facts and receiving knowledge in the broadest sense. Students saw research as a possibility to delve into a topic and learn academic skills at the same time.

I think it [research] is very interesting and I see this as a part of my academic training, which is missing in general medical training in my opinion. – S3

Subsequently, students also mentioned that they would be motivated to do research to comply with their personal needs like their *curiosity*, *need for challenge*, and need for *variety*.

I just want to have some extra challenge, because medical training on itself is just learning, learning, learning. And if you have something next to that more directly linked to practice and you see where you can end up, that motivates me. – S13

Moreover, students felt the need to *contribute to knowledge and patient care*. They mentioned that it would be motivating for them to conduct research if their research actually meant something for science or healthcare. Students described the process of creating or revising knowledge as motivating, but they mostly elaborated on what research could mean for patients. They related research to, for instance, helping more patients, and finding cures for diseases. These outcomes of research were highly motivating for students.

Especially when I hear that some things are still unknown, where no solutions are available, for instance multiple sclerosis (MS). My aunt has MS, and to see her like that every day, not being able to walk… and that there is no solution for that. In my opinion, there needs to come a cure for that. – S4

Students also mentioned that *different parts of conducting research* seemed fun, which in turn motivated them to conduct research. They said they especially liked seeing and creating results. Moreover, content was important and the writing process was very appealing to them. The social aspects of research, like *collaboration*, were motivating as well.

Especially the collaboration with others appeals to me, I like to collaborate with others. And the results at the end, that you made something beautiful together what turns out to be a big part of your career. – S4

Furthermore, reading or hearing about research related work of others and their enthusiasm is inspiring for students (i.e. *inspiring role models*) and contributes to their motivation for research.

I had a chemistry teacher and he investigated a very specific topic, a specific protein. And he was so, well a specialist I suppose, very enriched, that he could transfer that in a beautiful way. And actually, I was kind of, very, impressed with that […]. I can get inspired by that. – S10

Students also described the importance of research bringing them external rewards, such as *acknowledgments*. Students wanted to be able to show that they actually did research and mentioned publications as a possible reward of, and thereby motivating factor for, research. Furthermore students wanted opportunities to build a network and to distinguish themselves from others, and were motivated for research because it could help them in their future career steps, like securing a competitive residency spot.

I think that it depends on what kind of specialism I want to get in. And what is expected of you with regard to research. I have to be honest, it is not a really romantic reason, but yes… – S1

### Which factors contribute to demotivation for conducting research?

Students especially focused on the *content* of research itself and different demotivating parts of conducting research. For instance, research topic could play a large part in demotivating students to conduct research.

With regard to content, it could demotivate me very much I think. Imagine that this is a topic I am not very curious about, I think when I delve into it, really in detail, that I lose all my curiosity. – S1

Furthermore, in a broader sense, students found the difficulties of doing research demotivating. Students especially mentioned *processing of data* and *statistics* as uninteresting. These activities within research could really hold students back in their possible choice to conduct research.

All that gathering of data, SPSS. It has become something I fear […]. I think it is terrifying that I don’t know where to begin. – S12

It would also be demotivating for students when their contribution to both research and society is small, for instance when their research is not used in practice. Furthermore, students acknowledged that *disappointing results* are plausible, but at the same time they strongly felt like this would demotivate them for conducting research.

Moreover, students described a *lack of autonomy* as demotivating. Especially when students have no choice in what kind of research they perform and when students have to comply to a variety of rules, they did not want to conduct research.

When research would be imposed, than I really would not, like here is a topic, go do your research. That would be very demotivating. – S8

At the same time, a *lack of support* could be demotivating as well. Students did not want to have the feeling they are doing research alone. It seems like a balance between autonomy and support suits students best. Subsequently, students mentioned an *inadequate atmosphere or collaboration* within the research group to be very discouraging as well.

When I would be part of a research group with a very bad atmosphere, or when people are not willing to answer a question or help you, that seems very demotivating to me. And that has nothing to do with the research itself, but really the collaboration […]. So I think, mainly, when having the feeling you are alone, without the possibility to call for help, that seems very difficult to me. – S7

## Discussion

We qualitatively explored first-year students’ perceptions of research. Furthermore, we determined motivating and demotivating factors for conducting research. The pre-clinical students differed greatly in their perceptions of and motivation for research, which resulted in rich data with many different aspects. Within this data, some tensions emerged. On the one hand, students were able to describe important steps within the research process. On the other hand, students did tend to emphasize that certain parts of the research process, such as gathering of data and statistical analyses, were not appealing to them. Moreover, students perceived research as useful for clinical practice and personal development. However, students seemed to have negative perceptions in terms of what conducting research actually entails, and emphasized its difficulties and negative aspects.

In-depth analysis elucidated a variety of higher-order themes related to perceptions of research. In contrast to our results, a previous study of third-year medical students’ perceptions concluded that students had a narrow definition of research in the beginning of their third year [14]. Our results illustrate that *first-year undergraduate students* can already have broad perceptions of research. A possible explanation for this could be that an authentic learning situation at the beginning of medical training in which pre-clinical students conduct a small research project contributes to students’ knowledge of what research entails and its possibilities for clinical practice [22]. This is in line with the study by Imafuku and colleagues, showing that students’ initial narrow definition of research was somewhat broadened after their first research experiences [14].

**Fig. 1:**
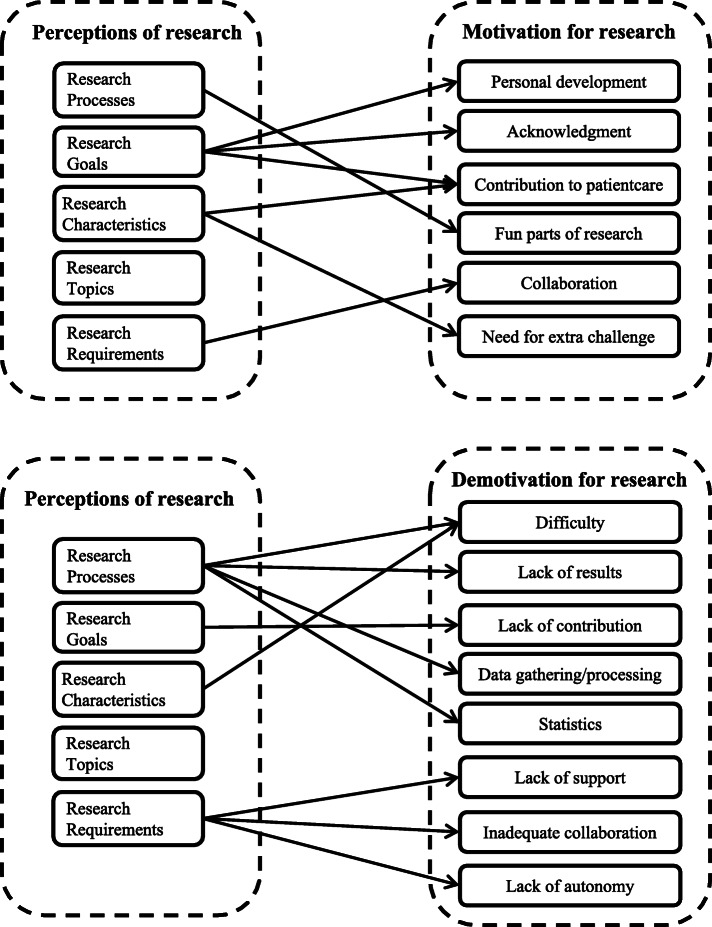
Main themes regarding student perceptions of research and its relations with motivating and demotivating aspects of conducting research

Going beyond our research questions, our results suggest a relation between perceptions of and motivation for research (Fig. 1). This is, among others, illustrated by students’ elaboration on various research goals, mainly focusing on its direct association with clinical practice and patient care. For instance, students viewed research as a way to make progress, develop medicine, create better physicians, and improve patient experiences. This direct association with practice contributed to students’ assumption that research is useful, emerging as a sub-theme of research characteristics. Additionally, these kind of topics were also identified by students as motivating, resulting in the theme ‘contributing to knowledge or patient care’ (Appendix 2). This implies that the social value of research is also something that could motivate students to subsequently conduct research. Therefore, medical schools may create conditions to raise awareness of the usefulness of research for clinical practice early in the curriculum. This could help pre-clinical students to develop positive perceptions of and motivation for research in early stages of medical education.

Nonetheless, there also seems to be a relation between perceptions and demotivation to conduct research. For instance, students tended to think that the biggest part of conducting research entails processing data and performing statistical analyses. Moreover, processing data and statistics also emerged as two subthemes of demotivating factors. This contributed to their idea that research is performed within a unilateral work environment (Appendix 2).

Previous studies showed that student perceptions of research are open to change [14, 26]. By targeting and adjusting unrealistic perceptions, such as the notion that research is merely statistics, motivation for research can be influenced. By acquainting pre-clinical students with the broader nature of conducting research, their perceptions can be altered. For example, students explicitly mentioned that writing is a fun aspect of research that contributed to their motivation. Therefore, educators could explicitly mention that this is part of the research process as well and that writing relies on creating results, for which statistical analysis could be necessary. Furthermore, statistics is unknown for many students and may seem frightening. Students are more inclined to pursue an activity when they feel confident about their capability in that domain (i.e. self-efficacy), and mastery of an activity leads to higher self-efficacy beliefs [27]. Students in pre-clinical phases of medical training lack experience with statistical analyses. Making statistics less ambiguous could also be a solution to motivate more students for conducting research. By letting students apply statistics directly to authentic research questions, even in their first undergraduate year, they can experience the relevance of statistics for creating results and finding answers to important questions. Through repeated practice with statistics, they can master it and self-efficacy beliefs may be enhanced.

Despite the grounded theory approach, parallels between the outcomes of our study and existing theories were visible. When students mentioned perceptions of research that also emerged as motivating or demotivating factors, they already gave an evaluation, connecting a favorable or unfavorable qualification to their perception. This is, for example, illustrated in perceptions of research as primarily being statistics, which students saw as a negative aspect. This seems to be in line with and substantiated by the Theory of Planned Behavior (TPB). TPB states that attitudes are a prerequisite for motivation, which in turn is related to certain behaviors. According to TPB, attitudes are perceptions of a certain behavior including the evaluation of the behavior (i.e. favorable versus unfavorable) [28]. This lends support to the idea that perceptions linked to motivation within our data are equal to ‘attitudes’ mentioned as an antecedent for motivation in TPB. Consequently, this also provides evidence for the idea that if perceptions of research are changed, motivation can be influenced as well. In turn, this offers opportunities to develop interventions and implement evidence-based strategies aiming to target student perceptions to motivate more students for research in early stages of medical school.

**Fig. 2:**
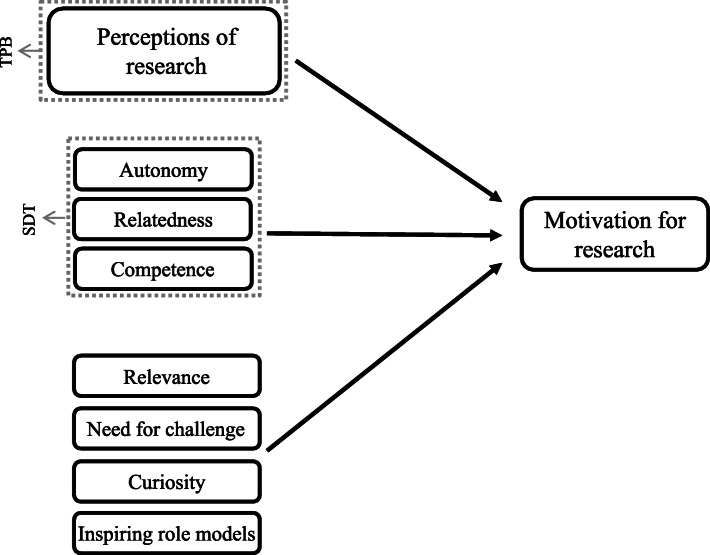
Prerequisites of motivation according to TPB and SDT, added by prerequisites as identified in our study

Our findings regarding autonomy, support, and development that are a necessity for student motivation are in accordance with and substantiated by the Self-Determination Theory (SDT). SDT states that motivation is influenced by three basic psychological needs: autonomy, relatedness, and competence [29]. These basic psychological needs are in line with the themes that emerged from our data. However, our data imply that influencing motivation entails more than only autonomy, relatedness, and competence (Fig. 2). A sense of relevance, e.g. being able to contribute to patient care, seems to have a major influence on motivation as well. Moreover, need for challenge and curiosity were also named as motivational factors. In addition, inspiring role models could be prerequisites for motivation as students emphasized they were inspired and became motivated by the work of others. Not only by reading scientific articles, but also by hearing about research related work from enthusiastic researchers. This provides insights in practical implications, as many educators conduct research as well and can communicate their own work in an enthusiastic way towards students during lectures or seminars. Providing students with opportunities to read articles and get acquainted with work of others seems to be a good possibility to contribute to their motivation as well.

When looking at our data, neither TPB nor SDT seem to comprehend all prerequisites for motivation. Hence, our study could contribute to the expansion of existing motivational theories like TPB and SDT, as illustrated in Fig. 2.

### Practical implications

In order to answer the fundamental question how conditions can be created under which students develop positive perceptions of and motivation for research in early stages of medical school, the emerged themes within the motivating and demotivating factors play a crucial role. Next to embedding research related courses in the curriculum and using educators as inspiring role models, our study provides other practical implications as well. Based on our results, it seems beneficial to create conditions in which students experience autonomy and the ability to work independently. In order to motivate students to conduct research, this seems to be key. Therefore, providing students with research experiences should be designed in such a way that students feel they are in control of their own research projects. Practically, this could be done by giving students multiple options regarding, for instance, the topic of their research. Furthermore, students could be stimulated to take a leading role in the implementation of their research. This not only contributes to feelings of autonomy, but is also related to the effective educational approach of ‘learning by doing’ as has been advocated by many throughout the years [30–35]. This is also reflected in our results, as our pre-clinical students mentioned that they would be motivated for research if they get the opportunity to actually perform research themselves. This stresses the need for more active learning approaches, providing students with research experiences in authentic learning situations in order to motivate more students for research.

Students were also in need of collaboration and wanted the possibility to rely on more experienced researchers. An inadequate atmosphere and lack of support are demotivating factors for students. This indicates the need for a balance between autonomy and support. In practice, this could mean that conditions need to be created in which students are able to become leaders of their research project, while a more experienced researcher closely monitors their development and provides support when needed. Furthermore, students indicated they were motivated when there were possibilities to develop competencies and receive acknowledgment or rewards. It would be beneficial to offer students the chance to work on their learning goals and mastery of research activities. Moreover, stimulating them to present their work in the form of publications or presentations at scientific meetings could enhance motivation for research and confidence [36]. In this way, students feel acknowledged for their work and are able to build a network. This should be embedded within education and explicitly communicated to students.

### Limitations and strengths

This study was conducted in one medical school, which may have implications for generalizability to other contexts. However, to the best of our knowledge, our study is the first to address perceptions of and motivation for research among medical students in early phases of medical training. We used qualitative methodology with an open and grounded approach, which is why we believe we elucidated actual student perceptions without steering towards certain outcomes. Furthermore, we applied thorough purposive sampling by using data of the same cohort of students in an earlier administered questionnaire in order to select a representative and diverse sample. We believe that these measures contributed to the great amount and variety of data in our study. Our findings provide new insights in the way beginning medical students perceive research, as well as factors promoting their motivation to conduct research. The findings contribute to both theory and practice, and may provide guidance for future quantitative research in which the generated hypotheses can be tested. Moreover, our results are in line with multiple existing theories. Therefore, we expect that our results may be applicable to other situations (e.g. educational programs within other countries, (post)graduate medical students) and may apprise education and studies in other contexts.

### Future research

It would be beneficial to study perceptions of and motivation for research in different educational programs and contexts in order to provide even more insights into how students’ positive perceptions and motivation for research could be promoted. Also, it would be an interesting future research avenue to conduct this study among medical students in other countries. Furthermore, it would be interesting to investigate the development of medical students’ perceptions of and motivation for research during medical training, in which they gradually engage in clinical practice. Our data suggested a relation between perceptions of and motivation for research, future research could be undertaken to investigate this hypothesis.

## Conclusions

Our study demonstrated that first-year students have broad perceptions and definitions of research. Additionally, a broad range of motivating and demotivating factors to conduct research were identified. Our results contribute to the idea that perceptions of research are related to motivation for research, which offers possibilities for interventions and promoting motivation for research through student perceptions. Furthermore, we identified relevance, curiosity, need for challenge, and inspiring role models as prerequisites for motivation in addition to perceptions as stated by TPB and autonomy, relatedness, and competence as stated by SDT. Consequently our study may contribute to expanding existing motivational theories like TPB and SDT. Moreover, conditions were identified under which pre-clinical students develop positive perceptions of and motivation for research during early phases of medical school in order to engage more students in research and make the first step to cultivate future physician-scientists.

## Data Availability

The data used and/or analyzed during the current study are available from the corresponding author on reasonable request.

## Appendix 1

### Interview Guide

Interview guide exists out of 3 topics: background, perceptions of research, and motivation for research. These three subjects will be discussed within the interview. The topics are comprised of numbered questions, which will be asked to start the interview and discussion with the individual students. The sub-questions will only be used when a student does not seem to understand the questions or does not know what to answer (which almost never occurred during the actual interviews).

1. **Background**
  1. What is your educational background?
  2. Why did you chose Medicine?
  3. Do you have previous experiences with research? If yes, could you elaborate?
2. **Perceptions of research**
  1. How do you perceive conducting research?
    Sub-question:
    - *What are the activities of a researcher*
    - *What are the abilities you should have to perform research*
    - *What can you do with research?*
    - *For whom is research important?*
  2. To which extent do you believe that research can be used as a physician? And in what way do you think physicians can use research?
    Sub-question:
    - *Should physicians use research in clinical practice?*
    - *Should physicians conduct research?*
3. **Motivation for research**
  1. Are you planning to conduct research yourself?
    If yes:
    What motivates you to conduct research?
    If no:
    What demotivates you to conduct research?
    If unknown:
    Ask what could motivate or demotivate to conduct research hypothetically
  2. Elaborating on the counterpart of the first question

### Appendix 2

**Table 1:** Overview of all emerged themes and sub-themes

Themes	Sub-themes
*1* Research processes	*1.1* Create research questions
	*1.2* Come up with methods
	*1.3* Gather data
	*1.4* Process data
	*1.5* Create results
	*1.6* Draw conclusions
	*1.7* Report
*2* Research goals	*2.1* Create new knowledge or refine existing knowledge
	*2.2* Solve problems
	*2.3* Answer questions
	*2.4* Find a given fact or pattern
	*2.5* Progress in science and healthcare
	*2.6* Development and improvement of medicines
	*2.7* Development and improvement of illness treatment
	*2.8* Better physicians
	*2.9* Improve work experience of physicians
	*2.10* Improve patient experience and trust
	*2.11* Improve organisation within the hospital
	*2.12* Intellectual development of physician-scientist
	*2.13* Prestigious for the career development of the physician-scientist
	*2.14* Improve education
*3* Research characteristics	*3.1* Hard
	*3.2* Detailed and careful
	*3.3* Intensive
	*3.4* Challenging
	*3.5* Large scaled
	*3.6* Useful
	*3.7* Additional obligations
	*3.8* Unilateral work environment
*4* Research topics	*4.1* Healthcare
	*4.2* Prevention
	*4.3* Organizational
*5* Research requirements	*5.1* Collaboration
	*5.2* Finance
	*5.3* Ethical approval
*6* Motivating factors	*6.1* Personal development
	*6.2* Acknowledgment or rewards
	*6.3* Contributing to knowledge or patient care
	*6.4* Curiosity
	*6.5* Different fun parts of conducting research
	*6.6* Variety
	*6.7* Ability to work independently
	*6.8* Topic
	*6.9* Opportunity to network
	*6.10* Possibilities to conduct research available
	*6.11* Research orientation
	*6.12* Collaboration
	*6.13* Inspiring role models
	*6.14* Need for extra challenge
*7* Demotivating factors	*7.1* Content
	*7.2* Other priorities
	*7.3* Lack of time
	*7.4* Mental pressure
	*7.5* Lack of support
	*7.6* Inadequate atmosphere or collaboration
	*7.7* Lack of or disappointing results
	*7.8* Lack of contribution
	*7.9* Difficulty
	*7.10* Gathering and processing of data
	*7.11* Statistics
	*7.12* Less attractive than clinical practice
	*7.13* Lack of autonomy
	*7.14* Misfit with personality

## Abbreviations

LUMC: Leiden University Medical Center
IRB: Institutional Review Board
TPB: Theory of Planned Behavior
SDT: Self-Determination Theory
